# Prevalence and risk factors of food insecurity among Syrian refugees in Türkiye

**DOI:** 10.1186/s12889-024-19129-x

**Published:** 2024-06-30

**Authors:** Kübra Esin, Tülay Işık, Feride Ayyıldız, Mustafa Koc, Hassan Vatanparast

**Affiliations:** 1 Department of Nutrition and Dietetics, Tokat Gaziosmanpaşa University, Tokat, Türkiye; 2 PhD Programs Nutrition and Dietetics, Istanbul Medipol University, Istanbul, Türkiye; 3 Department of Nutrition and Dietetics, Gazi University, Ankara, Türkiye; 4 Department of Sociology, Toronto Metropolitan University, Toronto, ON Canada; 5 School of Public Health, University of Saskatchewan, Saskatoon, SK Canada

**Keywords:** Syrian refugees, Food insecurity, Income, Employment

## Abstract

**Background:**

Although Türkiye (Turkey) hosts the largest number of Syrian refugees, studies on food insecurity are limited. This study examined the prevalence and risk factors of food insecurity among Syrian refugees living in Istanbul, which has the highest number of refugees in Türkiye.

**Methods:**

A cross-sectional survey was conducted among Syrian refugees in Istanbul between September 2021 and March 2022. The main income earners of 103 households were interviewed by a research dietitian, with the assistance of an Arabic speaking interpreter through hour-long face-to-face. Data on sociodemographic characteristics (age, gender, nationality, marital status, educational status, the family income, the major source of family income, and the number of family members living in the household etc.) and household food insecurity status were collected. Household food insecurity status was assessed with the eighteen-item Household Food Security Survey Module.

**Results:**

The household food insecurity rate was 90.3%, and those of adults and children were 88.4% and 84.8%, respectively. It was observed that family income level was significantly associated with food insecurity. A one-unit increase in monthly income increased food security by 0.02 times (*p* < 0.001). The number of employed refugees in the food security group was higher than that in the food insecurity group (*p* = 0.018). A significant difference was found in the rate of occupation type of the major income earner between the groups (*p* = 0.046).

**Conclusions:**

High rates of food insecurity, particularly severe food insecurity, were found among Syrian refugees living in Istanbul. While more research is warranted to explore the root causes and efficacy of the current support system, it requires the immediate attention of policymakers at the national and international levels to implement effective policies and interventions.

## Background

According to a report by the United Nations High Commissioner for Refugees (UNHRC), wars, violence, and persecution have led to the displacement of people at numbers unprecedented throughout history [1]. The civil war in Syria has led to one of the world’s greatest humanitarian crises, forcing approximately 5.3 million people to seek asylum in other countries [1, 2].

Türkiye (Turkey) hosts the greatest number of refugees and asylum seekers in the world and is one of the leading countries that has been affected the most by mass migration because of the crisis in Syria [3]. In this process, Türkiye has applied an open-door policy, and as a result, it has faced mass migration that is intense and difficult to keep under control. Syrian nationals as well as refugees and stateless individuals from Syria, who have arrived in Türkiye in masses or individually, have been granted temporary protection status [3]. Temporary protection was developed as an immediate solution in the event of a mass influx of refugees [4]. As of November 2023, Türkiye had granted this status to approximately 3.246.178 Syrians [5]. The Temporary Protection Regulation covers a range of rights, services, and assistance for the beneficiaries of temporary protection. These include access to healthcare, education, social assistance, psychological support, and access to the labor market [6].

Although some Syrians with a temporary protection status have returned to the secured regions of their country, a large number of them remain in Türkiye due to the continued domestic unrest and war in their country. Other factors for remaining in Türkiye includes social, economic, and political reasons such as better living and working conditions, and getting citizenship in Türkiye [7].

Having enough food is one of the basic needs that refugees have difficulty with. Many factors, ranging from economic and cultural differences in the host country to on-camp and out-of-camp settlements, affect the nutritional status of refugees [8, 9]. It is known that refugees living in camps have better access to healthy nutrition [10]. Even so, only 1.4% of Syrians in Türkiye live in refugee camps [5]. Refugees who live outside of camps reside in crowded houses with other refugees, often with 6–8 individuals in a household, on average [11]. In this case, not only their home but also things such as food are shared. To ensure and sustain a healthy life, access to sufficient and healthy nutrition and living conditions are essential [12].

Food insecurity is defined as “limited access to or the unavailability of nutritionally adequate, culturally relevant, and safe food and/or limited or uncertain ability to acquire food in socially acceptable ways” [13]. Food insecurity is a key social determinant of health. Social factors that lead to food insecurity are health status, employment status, income level, and refugee status. In this sense, refugees and asylum seekers constitute one of the high-risk categories in terms of food insecurity [14, 15]. This is because they mainly rely on less-expensive food mostly carbohydrates and fats due to economic problems and challenges with accessing healthy foods, and they eat just one or two meals a day [16]. Such dietary patterns lead to health problems such as obesity, diabetes, and heart disease [16, 17]. Additionally, serious problems such as vitamin deficiency, anemia, stunting in children, and severe malnutrition related to insufficient and unbalanced diets are associated with food insecurity [18, 19]. Determining food insecurity and ensuring food security for refugees and asylum seekers is one of the key concerns in terms of preventing and treating health problems caused by displacement [10]. Despite the increasing need for representative data on the health and food insecurity status of refugees, they are underrepresented in public health research [20]. Refugees have been considered “hard-to-reach” populations by some international experts because they have limited access to or excluded from healthcare systems, their geographical location, socioeconomic situation, high mobility, distrust of institutions, and language and cultural gaps [21].

Although Türkiye hosts the largest number of Syrian refugees, studies on food insecurity are limited [22, 23]. Research on refugees by obtaining reliable scientific data is vitally important and necessary for developing strategies and policies to address their specific needs [24]. This study examining the prevalence of and risk factors for household food insecurity among Syrian refugees in Istanbul, which has the highest number of refugees in Türkiye.

## Methods

### Study design and population

This cross-sectional study was conducted in Istanbul, where the highest number of Syrian refugees live [5]. The sample size was determined as 277 individuals with 5% deviation and 5% margin of error with a statistical power of 80% [25]. Assuming that there are at least three individuals in each household, we aimed to reach at least 100 households. Thus, interviews were held with the individuals of 103 households. Syrian refugees with temporary protection status, residence permits, Turkish citizenship, and no ID status living in Istanbul were included in the study. For brevity, we use ‘refugee’ in general but acknowledge the complex identities subsumed under these terms, such as asylum seeker, protected person, stateless person etc.

Snowball and purposive sampling methods are the most commonly used techniques when interviewing hard-to-reach populations [26]. Thus, the snowball sampling method was used in this study. Refugee organizations and society volunteers helped us to obtain access to appropriate Syrian refugees. The interviewed families then referred us to other families. Voluntary adult participants who lived in different areas of Istanbul were included in the study after they signed consent forms.

### Data collection

The study was conducted between September 2021 and March 2022. A research dietitian, with the assistance of an Arabic speaking interpreter, collected data through hour-long face-to-face interviews. The questionnaire used in the study consisted of two parts. In preparing the questionnaire form, the study protocol by Vatanparast et al. [27] was used. The first part consists of questions inquiring about the sociodemographic characteristics of the individuals, and the second part includes questions about their food security/insecurity status. In the first part, sociodemographic characteristics of the individuals, such as age, gender, nationality, marital status, educational status, the major source of family income, and the number of family members living in the household were inquired about. As the individuals hesitated to report their net income, it was asked if the family income was below or above the minimum wage of the period when the data were collected. The minimum wage is defined as “the wage paid to workers for a normal working day, which is sufficient to meet the minimum level of the worker’s compulsory needs such as food, housing, clothing, health, transportation, and culture at current prices” [28]. The questions were primarily directed to the breadwinner of the household, and when this was impossible, the questions were asked to other household members, preferably mothers.

Household food insecurity status was assessed with the eighteen-item Household Food Security Survey Module (HFSSM) [29]. HFSSM determines food insecurity at the household, adult, and child levels. This module consists of 18 questions, and by evaluating the individuals’ access to food in the last 12 months, their food insecurity risk is assessed. The first 10 questions evaluate food insecurity for adults, and the last 8 questions assess food insecurity for children. An Arabic validity and reliability study of the module questions was conducted for Syrian refugees [27].

The determination of adult, children, and household food security status based on the household food security survey module is given in Table 1 [30]. The food insecurity data were evaluated, and the participants were divided into groups as the group with food security and the group with food insecurity [25, 27].

**Table 1:** Determination of adult, child, and household food security status based on the household food security survey module

Food security status	10 item adult food security scale	8 item child food security scale	Household status
Food secure	No affirmative responses	No affirmative responses	Both adult status and child status are food secure
Marginal food insecure	No more than 1 affirmative response	No more than 1 affirmative response	Either adults or children, or both adults and children in the household are marginally food insecure and neither is moderately or severely food insecure
Moderate food insecure	2 to 5 affirmative responses	2 to 4 affirmative responses	Either adults or children, or both adults and children in the household are moderately food insecure and neither is severely food insecure
Severe food insecure	6 or more affirmative responses	5 or more affirmative responses	Either adults or children in the household are severely food insecure

### Statistical analysis

The data were analyzed using IBM SPSS Statistics for Windows 24.0 (IBM Corp., Armonk, NY, USA). Categorical variables were presented as the frequency (n) and percentage (%), and descriptive statistics for the continuous quantitative data were presented as the arithmetic mean (X̅) and standard deviation (SD). The Kolmogorov‒Smirnov test was used to check the normal distribution of the data. In the data analysis, the Mann‒Whitney U test was used to compare two nonparametric groups. For comparisons of the categorical variables, Fisher’s exact test was used. In the multivariable analysis, logistic regression was used. The Hosmer‒Lemeshow test was used to determine the goodness of fit of the logistic regression model. In the logistic regression analysis, the association between gender, monthly income, highest women’s educational level, income earner’s educational level, and duration of stay with food insecurity was evaluated. Statistical significance was accepted as *p* < 0.05 for all of the analyses.

## Results

The main income earners of 103 households were interviewed. Living in these households was 534 individuals, comprising 247 children and 287 adults. Additionally, living in 21.4% of the households (*n* = 22) were relatives in addition to the nuclear family. The average number of individuals living in these households was 5.1 ± 2.19, and their mean duration of residence in Türkiye was 7.4 ± 1.56 years. The prevalence of food insecurity among Syrian refugees living in Istanbul is presented in Table 2. The rate of household food insecurity was 90.3%, and those of adults and children were 88.4% and 84.8%, respectively.

**Table 2:** Prevalence of household food insecurity among Syrian refugees

	Children (*n*: 85)		Adults (*n*: 103)		Household (*n*: 103)	
	*n*	%	*n*	%	*n*	%
Food secure	13	15.3	12	11.6	10	9.7
Marginally food insecure	9	10.6	5	4.9	7	6.8
Moderately food insecure	27	31.8	26	25.2	20	19.4
Severely food insecure	36	42.3	60	58.3	66	64.1
Total food insecure	72	84.8	91	88.4	93	90.3

The relationships of food insecurity with sociodemographic variables are given in Table 3. In addition, 90% of those belonging to the food security category and 33% of those who were food insecure earned more than the minimum wage (*p* = 0.001). 100% of those with food security spoke Turkish at a fair-fluent level, while 29.0% of those with food insecurity spoke Turkish at a nonpoor level (*p* = 0.059). The proportion of refugees who were employed among those who were food secure was higher than that among those who were food insecure (*p* = 0.018). A significant difference was found in the rate of occupation type of the major income earner between the groups (*p* = 0.046).

**Table 3:** Relationship of household food insecurity with sociodemographic variables

Sociodemographic Characteristics	Food security status (*n*: 103)		*p*
	Food security (*n*: 10)	Food insecurity (*n*: 93)	
Age (years)*	28.5 ± 6.76	32.8 ± 10.02	0.230
Gender (%)**	F: 30.0%, M: 70.0%	F: 16.1%, M: 83.9%	0.374
Marital Status (% Married)**	70%	79.6%	0.689
Total number of individuals in the household*	4.7 ± 2.79	5.2 ± 2.10	0.448
Number of children in the household*	1.7 ± 1.76 (0–5)	2.4 ± 1.78 (0–9)	0.174
Total income (monthly)**			
< Minimum wage	1 (10.0%)	62 (66.7%)	**0.001**
> Minimum wage	9 (90.0)	31 (%33.3)	
Status in Türkiye**			
Temporary protection	8 (80.0%)	83 (89.2%)	0.329
Others	2 (20.0%)	10 (10.8%)	
Number of years of living in Türkiye*	7.4 ± 1.26 (5–9)	7.4 ± 1.60 (3–11)	0.860
Reasons for leaving Syria**			
War	8 (80.0%)	87 (93.5%)	0.173
Others	2 (20.0%)	6 (6.5%)	
Reasons for coming to Türkiye**			
Geographic proximity	5 (70.0%)	41 (44.1%)	
Business opportunities	2 (20.0%)	27 (29.0%)	0.182
Others	3	25(26.8%)	
Rate your Turkish language proficiency**			
None-Poor	-	27 (29.0%)	0.059
Fair-Fluent	10(100%)	66 (71.0%)	
Number of employees*	1.4 ± 0.51	1.0 ± 0.57	**0.018**
Current employment status of the major income earner **			
Employed full-time	7 (70.0%)	41 (44.0%)	
Employed part-time	3 (30.0%)	42 (45.2%)	0.323
Unemployed/Searching for a job	-	10 (10.8%)	
Education of the major income earner**			
< Grade 12	5 (50.0%)	67 (72.0%)	0.163
> Grade 12	5 (50.0%)	26 (28.0%)	
Occupation type of the major income earner**			
Worker	5(50.0%)	70 (75.3%)	
Officer	2(20.0%)	3 (3.2%)	**0.046**
Self-employed	3(30.0%)	12 (12.9%)	
Unemployed	-	8 (8.6%)	
Highest level of education of the women**			
< Grade 12	5 (50.0%)	67 (72.0%)	0.163
> Grade 12	5 (50.0%)	26 (28.0%)	

F: Female, M: Male

*Mann‒Whitney U test, **Fisher’s Exact test

Risk factors associated with the food insecurity status of the Syrian refugees in the sample of Istanbul are given in Table 4. The income level of the family was statistically significant in affecting food security. A one-unit increase in monthly income increased food security by 0.02 times.

**Table 4:** Factors associated with the household food insecurity status of Syrian refugees

Sociodemographic Characteristics	Odds Ratio (95% CI)	*p*
**Gender**		
Male	1	0.25
Female	0.36 (0.06–2.09)	
**Total income (monthly)**		
< Minimum wage	1	< 0.001*
> Minimum wage	0.02 (0.00-0.40)	
**Highest women’s education**		
Illiterate/Primary school	1	0.68
High School or Higher education	0.63 (0.07–5.41)	
**Income earners’ education**		
Illiterate/Primary School	1	0.50
High School or Higher Education	2.00 (0.26–4.20)	
**Duration of stay in Türkiye**		
> 5 years	1	0.28
≤ 5 years	5.27 (0.24–6.63)	
**Number of individuals**		
1–5	1	0.63
≥ 6	1.51 (0.27–8.35)	

*Model included: gender, income, women’s education, earner’s education, duration of stay in Türkiye, number of individuals

## Discussion

In this study, household risk factors and the frequency of food insecurity among Syrian refugees living in Istanbul were investigated. The rate of household insecurity was found to be 90.3%. The income levels of the participants were also determined to be significantly associated with their food insecurity status.

The high rate of household food insecurity observed in this study is similar to the results of studies conducted in Canada [25] and Lebanon [31]. In another study conducted in the Samsun Province of Türkiye, the rate of food insecurity was found to be 47.0% among Syrian refugees [22]. In a study that investigated food insecurity among Syrian refugees living outside of camps in Türkiye (Gaziantep, Hatay, Kilis, and Sanliurfa), the status of household food insecurity was analyzed by applying the World Food Programme (WFP) methodology. Overall, 30% of the households were food insecure, and 4% of these were severely food insecure [23]. Contrary to the results of several other studies [15, 23], in this study, almost two-thirds of the participants and their families were found to be severely food insecure (64.1%). This may have resulted from the higher cost of living, especially that of rent and food, in Istanbul. According to the regional distribution of household consumption expenditures in Türkiye, Istanbul was determined to be the region with the highest share of expenditures for housing (28.6%) [32]. The use of income for purposes other than food (such as rent and clothing) leaves limited funds for obtaining healthy food. The difference between these findings and the other studies mentioned above may have been due to differences in the methodological approaches used in the evaluation of food insecurity.

A low level of income was reported to be the main determinant of food insecurity [33]. In studies conducted on refugees, it has been demonstrated that as the household income level decreases, food insecurity increases [22, 25, 34]. Similarly, it was found in this study that household income level was statistically significantly associated with food security (*p* = 0.001). A one-unit increase in monthly income increased the food security rate by 0.02 times (*p* = < 0.001). In the case of income deficiency, families resort to methods of decreasing food consumption, such as reducing the number and portions of meals, consuming certain types of food, preferring cheaper foods, being hungry all day, and restricting the food of adults so that children can eat [35]. In this study, the food insecurity rate was higher in adults than in children, at 88.4% and 84.8%, respectively. In other studies, similar results were obtained [25, 36], and it was reported that a strategy used by families to cope with food insecurity was mostly adults, particularly women, restricting their own food consumption to provide food for their children [37].

It was reported that sociodemographic characteristics such as age, gender, marital status, and the duration of stay in the host country can affect food security status [38]. However, the results of studies examining the relationship between food security and the sociodemographic characteristics of refugees are contradictory [25, 39]. While Mansour et al. [39] found a relationship between sociodemographic characteristics and food security among Middle Eastern and North African migrants and refugees in high-income countries, Al-Kharabsheh et al. [25] found no relationship among them in Syrian refugees in Canada. In this study, although there were differences between the group with food security and the group with food insecurity in terms of the sociodemographic characteristics (age, gender, marital status, number of children in the household, total number of individuals in the household, and number of years living in Türkiye) in terms of percentages, these differences were not statistically significant. This may be because the majority of the sample (90.3%) were at risk of food insecurity. In addition, differences such as the ethnicity of the refugees, the host country, and the study methodology may cause differences in the results of the study.

The nutrition and health status of refugees can be affected by not only the socioeconomic conditions of the host country but also whether they live in camps [8, 9]. The majority of Syrian refugees in Türkiye prefer to live outside of the camps [5]. This means crowded households, including extended family members, leading up to 6–8 individuals living together [11]. In this study, the average number of individuals living in a household was 5.1 ± 2.19, while relatives other than the nuclear family lived in 21.4% of these households. Moreover, the number of employed refugees in the food insecurity group was lower than that in the food security group (*p* < 0.05). The low number of income earners in households can lead to economic insufficiency and the inability to access adequate food [40].

A refugee’s employment status is associated with their cultural adaptation to the host country, their ability to speak the language of the country, and their educational level [25]. Out of the 2.16 million Syrians of working age in Türkiye, only 1 million are estimated to participate in the labor market, most of them informally in low-skilled and low-paid jobs [41]. In this study, all of those with food security worked at a full-time or part-time job, while 10.8% of those with food insecurity were unemployed. Additionally, there was a significant difference in the rate of occupation type of the major income earner between the groups. As employment generates income, the relationship between employment status and food security status is predictable [42].

The inability to speak the language of the host country fluently is associated with lower employment opportunities and lower income among refugees [43]. Gingell et al. [44] stated that poor language skills were associated with household food insecurity, and this was often cited as a major barrier to food security for refugees. Although it is not statistically significant, in the present study, those who were fluent in Turkish had higher levels of food security than those who were not fluent. Previous studies have demonstrated that a low level of education, especially among women, is associated with higher food insecurity rates [45, 46]. However, there was no relationship between food security and education level in the present study. Similarly, Chevrier et al. [47] found no association between food insecurity and education level among Syrian refugees. This may have been as a result of the lack of university equivalence between the countries or the lack of recognition of the refugees’ education level in the host country if they did not have adequate documentation [44]. Therefore, there may not have been a difference according to gender or educational status.

### Limitations

Several limitations to this study need to be acknowledged. The first had to do with the household income level. Participants could not provide precise figures; they gave just rounded averages. Second, after initially agreeing to participate, some respondents declined for various reasons, which affected the sample size. Finally, carrying out the study in a metropolitan city may introduce selection bias and limit generalizability. We recommend longitudinal studies and mixed method trials with a larger sample of households in the future to determine the factors affecting food insecurity and coping strategies.

## Conclusion

In conclusion, considerably higher rates of food insecurity were found among Syrian refugees living in Istanbul. It was observed that family income level was significantly associated with food insecurity. The high rate of food insecurity observed among the refugees in Istanbul suggests the need for programs to enhance livelihoods and financial independence of refugees living in cities with a higher cost of living. Providing Turkish language courses to individuals who come from different countries and speak different languages will help them adapt to the culture and increase their job opportunities. One of the Sustainable Development Goals relevant to persons of concern to the UNHCR for 2030 is to end hunger, achieve food security and improved nutrition, and promote sustainable agriculture [48]. The synergetic efforts of governments, intergovernmental organizations, and nongovernmental organizations are needed to realize these goals and will contribute to a decrease in the incidence of food insecurity. Determining reasons for food insecurity, revisiting existing policies, and developing new policies is essential in terms of both protecting the health of refugees and asylum seekers, especially children and adolescents [49] and decreasing the costs related to healthcare for these individuals. It is hoped that the findings of this paper will provide a guide for future studies.

## Data Availability

The datasets used during the present study can be obtained from the corresponding author on reasonable request.
